# Theory on the Dynamics of Feedforward Loops in the Transcription Factor Networks

**DOI:** 10.1371/journal.pone.0041027

**Published:** 2012-07-20

**Authors:** Rajamanickam Murugan

**Affiliations:** Department of Biotechnology, Indian Institute of Technology Madras, Chennai, India; University of Zurich, Switzerland

## Abstract

Feedforward loops (FFLs) consist of three genes which code for three different transcription factors A, B and C where B regulates C and A regulates both B and C. We develop a detailed model to describe the dynamical behavior of various types of coherent and incoherent FFLs in the transcription factor networks. We consider the deterministic and stochastic dynamics of both promoter-states and synthesis and degradation of mRNAs of various genes associated with FFL motifs. Detailed analysis shows that the response times of FFLs strongly dependent on the ratios (*w_h_* = *γ_pc_/γ_ph_* where *h = a, b, c* corresponding to genes A, B and C) between the lifetimes of mRNAs (1/*γ_mh_*) of genes A, B and C and the protein of C (1/*γ_pc_*). Under strong binding conditions we can categorize all the possible types of FFLs into groups I, II and III based on the dependence of the response times of FFLs on *w_h_*. Group I that includes C1 and I1 type FFLs seem to be less sensitive to the changes in *w_h_*. The coherent C1 type seems to be more robust against changes in other system parameters. We argue that this could be one of the reasons for the abundant nature of C1 type coherent FFLs.

## Introduction

Transcription factors (TFs) regulate the quantitative levels of many proteins inside a cell [1]–[4]. TF networks consist of several fundamental building blocks such as auto regulatory loops, flip-flops, feedback loops, single input modules, cascades, feed-forward loops (FFL) and dense overlapping regulons [5]–[7]. Positive auto regulatory loops play critical roles in the maintenance of cellular memory [3] and reprogramming whereas a negative auto regulatory loop seems to speed up the response time against an external stimulus [8]–[9]. The response time of a gene/motif is the amount of time that is required to achieve half of the steady-state concentration of resultant protein product which is often referred to as rise-time [3]–[4].

**Figure 1:**
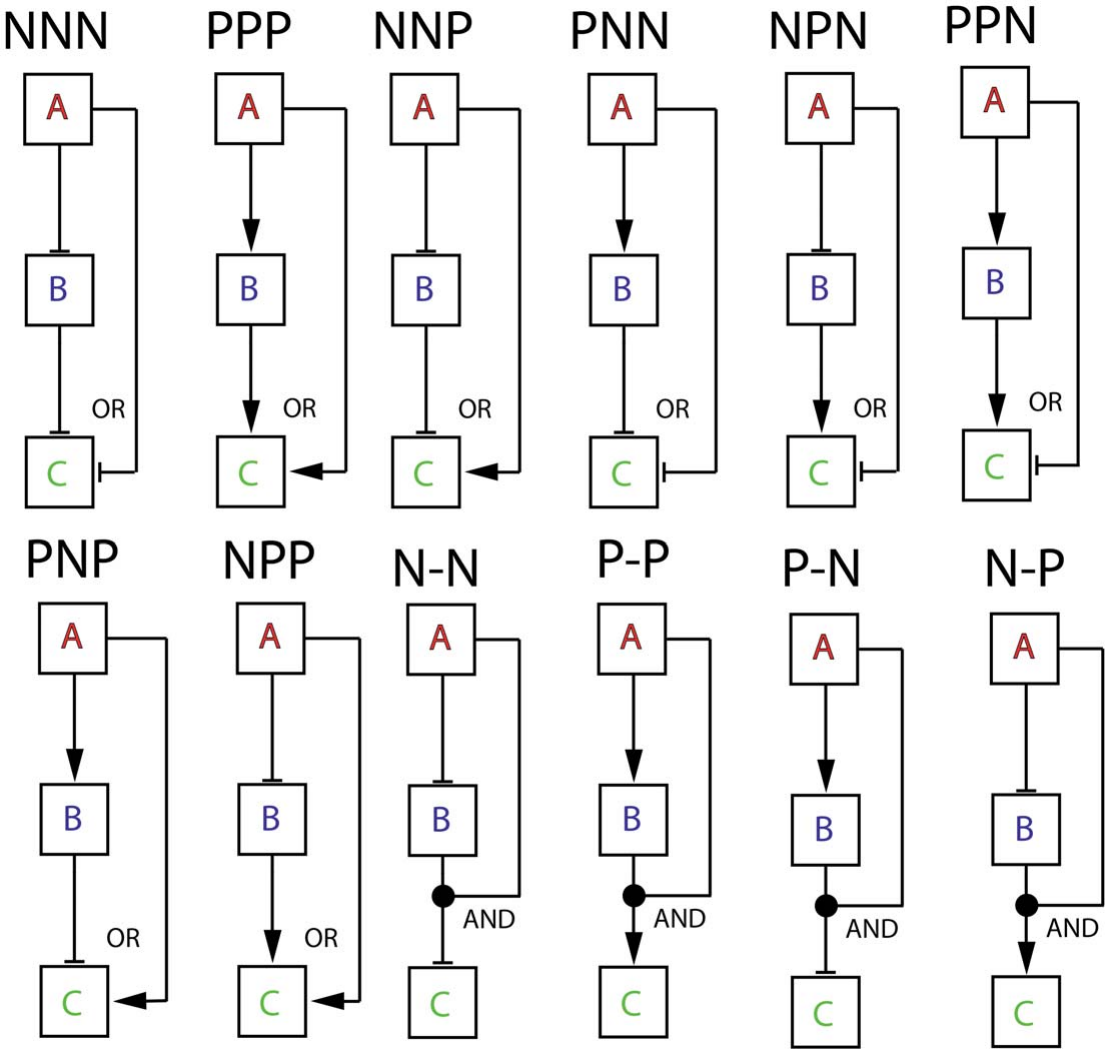
Various types of feedforward loops (FFLs) considerd. FFLs consist of three genes which code for three different transcription factors A, B and C where B regulates C and A regulates both B and C. There are three regulatory connections in FFLs. Since each of these regulatory connections can be either positive or negative, totally there are eight different FFLs. We use three letter codes as “FGH” where ‘F’ denotes the type of regulation of B by A and ‘G’ denotes the type of regulation of C by B and ‘H’ denotes the type of regulation of C by A. Here PPP, NPN, PNN and NNP (termed as C1, C2, C3 and C4) are coherent type and PNP, NNN, PPN and NPP (termed as I1, I2, I3 and I4) are incoherent type. When dimer of A and B regulate C through AND-logic, there are four possible FFLs as P-P, P-N, N-N and N-P.

Feedforward loops consist of three different genes namely A, B and C which code for three different TFs. Here the protein of gene A regulates the transcription of both B and C whereas both the proteins of genes A and B regulate the transcription of C (Fig. 1). As shown in Figure 1, totally there are three such regulatory connections in a FFL network motif and eight such regulatory combinations viz. (PPP, PNP, NNN, NPP, PNN, PPP, NNP, and NPN). The first one in a combination “FGH” denotes the type of regulation of the transcription of gene B by the protein of gene A, the second one stands for the type of regulation of C by B and the third one denotes the type of regulation of C by TF protein A. Here the type of regulation can be either positive or negative where P denotes positive and N denotes negative. Here PPP is classified as a coherent type FFL whereas PNP is classified as incoherent type. A FFL motif is said to be a coherent type if the direct effect of the general transcription factor (A) on the effector operons (C) has the same sign (negative or positive) as its net indirect effect through the specific transcription factor (B). Here PPP, NPN, PNN and NNP (termed as C1, C2, C3 and C4) are coherent types and PNP, NNN, PPN and NPP (termed as I1, I2, I3 and I4) are incoherent types [10]–[11]. Here A is the general transcription factor that directly regulates the effector operon of gene C and also indirectly regulates gene C through B. For example, in PNN coherent type, gene A positively regulates B which in turn negatively regulates C and therefore the net effect of regulation of C by TF gene A indirectly through B is negative. Since A directly regulates C via negative mode, PNN is called as a coherent type.

FFLs perform several important cellular tasks in various biological systems [11]–[15]. It has been shown that PPP type FFL can act as a sign sensitive delay [14]. For example when A and B regulate C via an AND type logic, then PPP type FFL shows a delay in the expression of gene C following induction of A by an external signal, but no delay following deactivation of A [14]. It seems that coherent types constitute ∼85% of the naturally occurring FFL motifs. Magnan and Alon [10] have comprehensively studied the dynamical and kinetic behavior of various types of FFLs under the condition that the promoters of both genes A and B were triggered by external stimuli/signals. Upon analyzing literature-based databases of experimentally verified direct transcription interactions for *E. coli* [6] and *S. cerevisiae* [7], they have discovered that PPP (C1) and PNP (I1) type FFLs are more abundant in nature than others [10]. Although all the FFLs are biologically feasible [10], it is still not clear why these two types were preferably selected by nature against other types in these organisms.

Most of the earlier theoretical and experimental studies on FFLs assumed a quasi-equilibrium condition for the binding-unbinding dynamics of regulatory TF proteins at various promoters [10] and a steady-state condition for the dynamics of synthesis and degradation of mRNAs. These assumptions are valid [9] only when the timescales associated with the synthesis and degradation of TF proteins are much slower (several orders of magnitude) than the timescales associated with the binding-unbinding of regulatory TFs at the respective promoters and synthesis and degradation of mRNAs. Recently the role of mRNA stability in tuning the kinetics of gene induction has been studied in detail by Elkon et.al [16]. It seems that the rapidity of induction negatively correlates with the stability of mRNAs. Further, the dynamics of mRNAs can be approximated to be in a steady state only when the ratio *w = γ_p_/γ_m_* ( = lifetime of mRNA/lifetime of protein) is closer to zero which is not true for most of the protein coding genes [9]. Here *γ_p_* and *γ_m_* are the decay rate constants associated with the respective protein and mRNA. In prokaryotic systems *w ∼* 0.1 and in eukaryotic systems such as yeast *w* seems to vary [17]–[19] approximately from 0.1 to 1 with a median of ∼0.3. The response times associated with various TFs in a given network are strongly dependent on *w* of the respective genes and the rate of protein production will be in turn dependent on the response time [9]. TF regulatory networks whose response times are close to or lesser than the generation time of the cell and also less sensitive to the variation in the values of *w* corresponding to the component genes are more desirable. In this paper using a combination of theoretical and simulation tools we will (a) formulate a detailed model of various types of FFLs that includes the binding-unbinding dynamics of regulatory TFs at various promoters and synthesis and degradation of mRNAs, (b) investigate the effect of variation in *w* and other system parameters on the response times and overall dynamics of different type of FFLs using the detailed model and (c) explain why some of the FFLs are more abundant in nature than others.

## Results

### Theoretical Formulation

Feedforward loops consist of three genes coding for transcription factors (TF) A, B and C (Fig. 1). The corresponding concentrations of mRNAs are (*m_a_*, *m_b_* and *m_c_*) and the concentrations of the protein products are *p_a_*, *p_b_* and *p_c_* all are measured in mol/lit (M). Corresponding steady-state concentrations are denoted as (*m_as_*, *m_bs_*, *m_cs_, p_as_*, *p_bs_* and *p_cs_*). In the absence of any regulation or when the promoters are turned-on completely, these steady state values will be (*m_hs_ = k_mh_/γ_mh_*, *p_hs_* = *k_mh_k_ph_/γ_mh_γ_ph_* where subscripts *h = a, b, c* denote the TF genes A, B and C respectively). Here the transcription rate of TF gene ‘*h*’ is *k_mh_* (Ms^−1^) and the respective translation rate is *k_ph_* (s^−1^). The decay rate constant associated with the mRNA of gene ‘*h*’ is *γ_mh_* (s^−1^) and the decay rate constant corresponding to the protein product is *γ_ph_* (s^−1^). Here subscripts *h, g = a, b, c* respectively denote gene A, B and C. The gene associated with the transcription factor A is controlled/triggered by external signal which may be an arbitrary time dependent pulse function (we denote this as 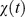) or an exponentially decaying one. There is a *cis*-acting element associated with the promoter of gene B where TF protein of A can bind and hence up/down regulate the expression of B via distal action that is mediated by either tracking or looping modes [20]. There are *cis*-acting elements associated with promoter of C where the protein products of both genes A and B or the dimer of A–B can bind and hence can up/down regulate C respectively in a “OR” or “AND” logic mode. In an A-OR-B mode the presence of either protein A or B is enough to up/down regulate the promoter of gene C. The protein products of TF gene A and B can also up/down regulate C in a “AND” type logic when the dimer of protein products A–B binds with the promoter of gene C. In this case the presence of both A and B is essential for up/down regulation of gene C. There are eight numbers of regulatory combinations with A-OR-B type logic and four different combinations are possible with A-AND-B type logic (Fig. 1). The fraction occupancy of promoter of TF gene ‘*h*’ by the respective regulatory TF protein ‘*g*’ is denoted as *X_hg_* 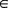 (0, 1) which is the ratio *x_hg_/d_hz_* where *x_hg_* is the cellular concentration of the promoter of gene ‘*h*’ that is bound with the protein of TF gene ‘*g*’ and *d_hz_* is the total concentration of the promoter of gene ‘*h*’ inside the cellular volume. There are at least two types of inter-molecular interactions [22] involved in the binding of TF proteins at the corresponding *cis*-regulatory sequences namely (a) a weak non-specific electrostatic interactions between the negatively charged backbone of DNA and the positively charged side chains of the aminoacids which are present at the DNA binding domains of TF proteins and (b) the specific hydrogen bonding interactions at the site-specifically bound DNA protein interface. The strength of electrostatic interactions will be modulated by the presence of water molecules at the DNA protein interface. In such well hydrated conditions at the DNA-protein interface, the net electrostatic interactions can be either attractive or repulsive owing to the presence of multiple electrical double layers around each charged group [22]. The free energy barrier associated with the fluctuating dynamics of TF proteins within this electrostatic field is comparable with that of the thermal free energy that in turn helps the TF proteins to freely slide along the DNA within this electrostatic capturing domain without physical dissociation [23]. Dissociation or unbinding (*X_hg_* = 0) of TF protein from the *cis*-acting site happens when all the specific hydrogen bonds are broken and the TF protein completely escapes from this electrostatic force field or capturing domain. When the TF protein is well within the electrostatic field then depending on the net inter-molecular interactions at the DNA-protein interface we find that *X_hg_* 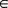 (0, 1). This is reasonable since there may be a situation where the site-specific hydrogen bonding network present at the interface of *cis*-regulatory DNA sequence and TF is broken due to thermal induced fluctuations but the TF protein is still present there within the electrostatic force field (partially bound condition). This partially bound promoter state is common in eukaryotic systems where a DNA loop connects the *cis*-acting module and promoter and holds the transcription initiation components so that they are nearby each other in three dimensional space within the electrostatic capturing domain. Further the binding-unbinding dynamics of TF protein ‘*g*’ at the promoter of TF gene ‘*h*’ will be observed as a continuous process in the timescale of the synthesis and decay of the corresponding mRNA and protein of TF gene ‘*h*’. This means that at the timescales of synthesis and decay of mRNAs and proteins, the thermally driven local fluctuations in the occupancy of the promoters by the TF proteins well within the electrostatic capturing domain will be averaged out. Under such conditions the averaged promoter state occupancy will be equal to the thermodynamic probability of finding the promoter to be occupied by the regulatory TF protein. Upon considering all these facts, one can conclude that it is appropriate to use a continuous type probability variable (such as *X_hg_*) to denote the promoter state occupancy to account for the promoter of gene ‘*h*’ that is partially bound with the TF protein ‘*g*’ rather than a discrete Boolean type variable as described earlier [9], [17]–[18]. The bimolecular collision rates associated with the binding of TF protein ‘*g*’ with the promoter of gene ‘*h*’ is denoted as *k_fgh_* (M^−1^s^−1^) and the corresponding off-rates of these bimolecular site-specific DNA-protein complexes are denoted as *k_rgh_* (s^−1^). *K_gh_* = *k_rgh_/k_fgh_* (M) is the overall dissociation constant corresponding to the site-specific binding of TFs at their respective cognate sites. We have summarized the parameters of our detailed model in Tables 1 and 2. Since there are several system parameters, exploration of the entire parametric hyperspace will be a complicated one. To simplify the calculations further we introduce the following scaling scheme to project the dynamical variables onto a dimensionless space.

**Table 1:** Definition of parameters used to describe the dynamics of various FFLs.

Parameter	Gene A	Gene B	Gene C	Units and remarks
*m_k_*	*m_a_*	*m_b_*	*m_c_*	M, conc. of mRNAs
*p_k_*	*p_a_*	*p_b_*	*p_c_*	M, conc. of proteins
*k_mk_*	*k_ma_*	*k_mb_*	*k_mc_*	Ms^−1^, transcription rate
*k_pk_*	*k_pa_*	*k_pb_*	*k_pc_*	s^−1^, translation rate
*γ_mk_*	*γ_ma_*	*γ_ma_*	*γ_mc_*	s^−1^, decay rate constant for mRNAs
*γ_pk_*	*γ_pa_*	*γ_pb_*	*γ_pc_*	s^−1^, decay rate constants for proteins
*m_ks_*	*k_ma/_γ_ma_*	*k_ma/_γ_ma_*	*k_ma/_γ_ma_*	M, steady state values of mRNAs in unregulated case
*p_ks_*	*k_pa_ k_ma/_γ_pa_γ_ma_*	*k_pa_ k_ma/_γ_pa_γ_ma_*	*k_pa_ k_ma/_γ_pa_γ_ma_*	M, steady state values of proteins in unregulated case
*M_k_ = m_k_/m_ks_*	*m_a_/m_as_*	*m_a_/m_as_*	*m_c_/m_cs_*	dimensionless
*P_k = _p_k_/p_ks_*	*p_a_/p_as_*	*p_b/_p_bs_*	*p_a_/p_cs_*	dimensionless
*w_k_ = γ_pc_/γ_mk_*	*γ_pc_/γ_ma_*	*γ_pc/_γ_mb_*	*γ_pc_/γ_mc_*	dimensionless
*ρ_k_ = γ_pc_/γ_pk_*	*γ_pc_/γ_pa_*	*γ_pc_/γ_pb_*	1	dimensionless
*d_zk_*	*d_za_*	*d_zb_*	*d_zc_*	M, total conc. of promoter
*λ_mh_*	*1/m_as_w_a_*	*1/m_bs_w_c_*	*1/m_cs_w_c_*	M^−1^, noise parameter
*λ_ph_*	*1/p_as_ ρ_a_*	*1/p_bs_ ρ_b_*	*1/p_cs_ ρ_c_*	M^−1^, noise parameter

**Note**: This table describes the variables and parameters used in the numerical simulations of different FFLs. Here *k = a, b, c* represent genes A, B and C respectively. The values *d_zk_* represent the concentration of promoters of the gene *k* inside the cell. The values *m_ks_* and *p_ks_* are the steady-state numbers of mRNA and protein molecules associated with gene *k* in the absence of any type regulation.

**Table 2:** Parameters used to describe the interactions between various components of FFLs.

Parameters	AB	BC	AC	Units and remarks
*σ_hk = _k_fhk_ d_zk_/γ_ph_*	*k_fab_d_zb_/γ_pa_*	*k_fbc_d_zc_/γ_pb_*	*k_fac_d_zc_/γ_pa_*	dimensionless
*K_hk_ = k_fhk/_k_rhk_*	*k_fab_/k_rab_*	*k_fbc_/k_rbc_*	*k_fac_/k_rac_*	M, dissociation constant connected with binding of protein ‘*h*’ with promoter ‘*k*’
*v_hk_ = γ_pc_/k_fhk_ p_hs_*	*γ_pc_/k_fab_ p_as_*	*γ_pc_/k_fbc_ p_bs_*	*γ_pc_/k_fac_ p_as_*	dimensionless
*µ_hk_ = K_hk_/p_hs_*	*K_ab_/p_as_*	*K_bc_/p_bs_*	*K_ac_/p_as_*	binding of protein ‘*h*’ with promoter of ‘*k*’
*x_hk_*	*x_ba_*	*x_cb_*	*x_ca_*	M, conc. of promoter ‘*h*’ bound with ‘k’ protein
*X_hk_ = x_hk_/d_zh_*	*x_ba_/d_zb_*	*x_cb_/d_zc_*	*x_ca_/d_zc_*	occupancy of promoter ‘*h*’ by protein ‘*k*’
*k_fhk_*	*k_fab_*	*k_fbc_*	*k_fac_*	M^−1^s^−1^, binding rate of ‘*h*’ with promoter ‘*k*’
*k_rhk_*	*k_rab_*	*k_rbc_*	*k_rac_*	s^−1^, dissociation rate of protein ‘*h*’ from promoter ‘*k*’

**Note**: This table describes various parameters associated with the different types of regulatory interactions in FFLs. The value *K_hk_* is the dissociation constant connected with binding of protein of gene ‘*h*’ with promoter of gene ‘*k*’. The column AB denotes the regulation of the promoter of gene B by the protein product of A and so on.



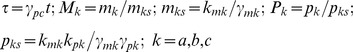
Here we measure the real time *t* in terms of numbers of lifetimes (1*/γ_pc_*) of the protein product of gene C. When protein C is stable over several generations of the cell, then its rise-time that is required to achieve half of the steady state value will be equal to the generation time of the cell upon considering the dilution owing to the doubling of cell volume along the process of cell division [8]–[9]. In such conditions one can also transform the dimensionless τ in terms of numbers of generation times of the cell by dividing as τ/ln2. With these definitions we can write the deterministic differential equations associated with temporal evolution of (*M_h_*, *P_h_* and *X_hg_* where *h = b, c* and *g* = *a, b, c*) of FFLs with A-OR/AND-B type regulatory logic imposed on the transcription of gene C as follows.

TF A:

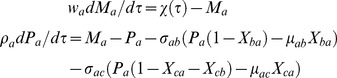
(1)The first one in Eqs 1 describes the dynamics of mRNA associated with the TF gene A whereas the second one describes the dynamics of protein synthesis, decay and binding-unbinding of protein A with the promoters of TF genes B and C. The dimensionless perturbation parameters in Eqs 1 are defined as follows.



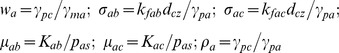
Here 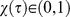 is a time dependent external signal that can turn on/off the expression of A. For a constitutive expression of TF gene A we can set 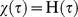 and depending on the type of the signals and their decay properties this function can be modified. To investigate the properties of the response-times of various types of FFLs we can set 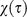 as rectangular pulses/dips with predefined widths. Here 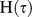 is the Heaviside step function such that 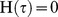 when 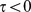 and 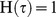 when 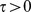. Rectangular pulses of signals at a given time point can be constructed with a combination of step functions. To introduce a rectangular pulse at the scaled time *τ = τ_p_* for a width of *θ*, we need to set 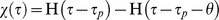. To generate a series of *n* numbers of rectangular pulses of signals at time points *τ_i_* with widths *θ_i_* where *i* = 1, 2…*n* we need to set 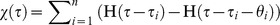. Further we assume that the basal expression levels of all TF genes A, B and C are zero.

TF B:

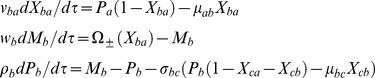
(2)The first one in Eqs 2 describes the binding-unbinding dynamics of TF protein A at the promoter of TF gene B. Second and third equations describe respectively the dynamics of synthesis and degradation of mRNA and protein products associated with TF gene B. The dimensionless perturbation parameters in Eqs 2 are defined as follows.



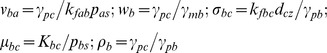
The function 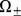 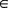
(0, 1) will vary depending on the type of regulation. For a positive regulation of the promoter of TF gene B by TF protein of A, we find 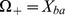 and for a negative type regulation we find 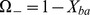.

TF C:

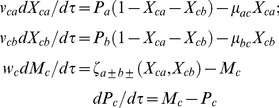
(3)The first set of equations in Eqs 3 describe respectively the binding-unbinding dynamics of TF proteins A and B at the corresponding *cis*-regulatory elements associated with the promoter of gene C. Second and third equations describe the dynamics of synthesis and degradation of mRNA and protein products of C. The dimensionless perturbation parameters in Eqs 3 are defined as follows.



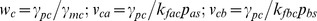
The function 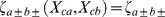 varies depending on the type of regulation. Here the total fraction of promoter of C occupied by the proteins of either A or B is *X_c_* = *X_ca_* + *X_cb_*. There are four different possibilities.
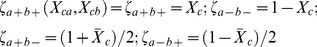
(4)Here the subscript “a+b+” indicates the case where both the TF proteins A and B positively regulate C and other combinations are defined in the similar way. In Eqs 4 we have defined 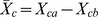 and depending on the type of regulation imposed on the promoter of gene C we find the following limiting conditions of the promoter-state occupancy of TF gene C.
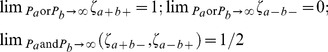
(5)Upon combining Eqs 1–4 we find that there are eight possible numbers of regulatory combinations as (PPP, NNN, PNP, NPN, NNP, PPN, NPP and PNN) in case of A-OR-B type FFL. Here PPP, NPN, PNN and NNP are coherent type FFLs (corresponding standard notations are namely C1, C2, C3, and C4) whereas PNP, NNN, PPN and NPP (corresponding standard notations are namely I1, I2, I3 and I4) are incoherent types FFLs. There will be an additional step corresponding to the dimerization of TF proteins A and B in case of FFLs with A-AND-B logic type regulation. The dynamics of protein-protein dimerization and dissociation can be described by the following differential equation.
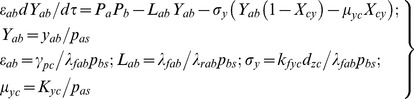
(6)Here yab is the cellular concentration (M) of the dimer of the protein products of TF genes A and B, λfab and λrab are the corresponding forward bimolecular (M−1s−1) and reverse unimolecular (s−1) rate constants associated respectively with the dimerization and dissociation reactions. To be consistent with Eqs 6 the equations associated with the dynamics of synthesis and degradation of Pa and Pb will be modified as follows.
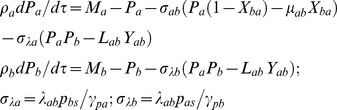
(7)Further when the dimer A-B binds with the promoter of gene C and hence up/down regulate then the related rate equations corresponding to the expression of gene C will be modified as follows.



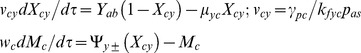
(8)The function 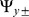 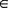 (0, 1) varies depending on the type of regulation of the promoter C by the A–B dimer. For a positive regulation we find 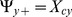 and for negative regulation 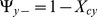. Upon combining Eqs 2 and 8 we find four different types of FFLs with A–AND–B type logic on the promoter of gene C viz. (P–P, P–N, N–P and N–N). Here one should note that P-P is similar to PPP type FFL however with A–AND–B gated logic at the promoter of TF gene C, and P-N corresponds to PNN type, N–P corresponds to NPP, and N–N corresponds to NNN. In a combination “K–H”, ‘K’ is the type of regulation of promoter B by protein A and ‘H’ is the type of regulation of promoter C by A–B dimer.

### Steady-state Analysis

When the dynamics of the variables *X_hk_* and *M_k_* are much faster than the rate of synthesis and degradation of the corresponding proteins *P_k_* then we have the following limiting conditions for A-OR-B regulation.
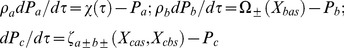
(9)Eqs 9 can be obtained by setting *w_k_* = 0 for all *k = *(*a, b, c*) and *v_b_ = v_ca_ = v_cb_* = 0 in Eqs 1–3. The steady state values of the protein products *P_hs_* where *h = a, b, c* will vary depending on the type of regulation and protein-protein interactions between A and B. Here we have defined the steady state values of *X_hk_* in the coupled Eqs 9 as follows.



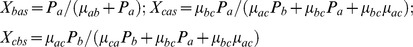
Similarly one can derive the limiting condition in the presence of A-AND-B type regulation of promoter C by the dimer of the protein products of genes A and B. In such conditions the differential equation associated with *P_c_* in Eqs 9 will be modified as follows.
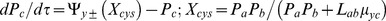
(10)Here one should note that the dynamical variables (Pa, Pb and Pc) also represent the efficiency of TF genes A, B and C in raising their protein levels toward their unregulated steady state values (phs = kmhkph/γmhγph where h = a, b, c) even in the presence of various types of positive/negative type regulation on their promoters. The maximum achievable steady state values of Phs will be Phs = 1. In case of PPP type A-OR-B FFL, Pa and Pb influence the rate of change in Pc in an additive way. Whereas in case of P-P type A-AND-B FFL Pa and Pb influence the rate of change of Pc in a multiplicative way. As a result of this multiplicative effect, P-P type FFL can effectively filter out the short/transient pulses of signals which are introduced at the promoter of TF gene A. When TF proteins A and B decay much faster than TF protein C, then we find that as 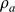 and 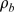 tend toward zero. Under such conditions, the expression for Xcys in Eqn 10 can be written as follows.
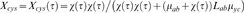
(11)Eqn 11 suggests that when the binding parameters (µab, Lab, µyc) are much lesser than one and the promoter of gene A is turned on for sufficiently longer time periods then the TF gene C will be turned on to its maximum expression level since Xcs∼1 under such conditions. Similar to Eq 11 when 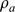 and 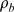 tend toward zero, then we can write the expression for the occupancy level of the promoter C by TF proteins of genes A or B in case of PPP type A-OR-B FFL as follows.
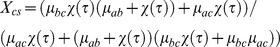
(12)This equation suggests that when the binding parameters (µbc, µac) are much lesser than one and the promoter of gene A is turned on for sufficiently longer time periods, then the TF gene C will be turned on to its maximum expression level since 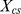
∼ 1 under such conditions. Using Eqs 11 and 12 one can derive the steady-state values (Pas, Pbs, Pcs) of scaled protein concentrations Pa, Pb, and Pc associated with Eqs 9 as follows.
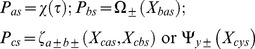
(13)To evaluate Pbs one needs to substitute 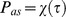 in the appropriate expression of 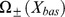 that in turn depends on the type of regulation of promoter of the TF gene B by the protein of gene A and subsequently these Pas and Pbs need to be substituted in the appropriate expression of Pcs that depends on the types of regulation of TF gene C by proteins A and B. While deriving Eqs 1–3 related to A-OR-B type FFLs we have assumed that monomeric units of proteins A or B interact with the promoters of B and C. Similar to the regulation of C by the dimer of TF proteins A–B in case of A-AND-B type FFLs, one can generalize Eqs 1–3 corresponding to A-OR-B type FFLs to include the regulation of the promoter of the TF genes B and C by the multimeric form of TF proteins A and B respectively [10]. So for we have set wk = 0 for all k = a, b and c. One can show that the response-time associated with the synthesis of the terminal TF protein C upon induction of the promoter of TF gene A by an external signal is strongly dependent on wc as follows. Consider a quasi-equilibrium situations for the promoter state occupancies and steady state situation for the synthesis and degradation of TF genes A and B so that (wa, wb) = 0 and (vca, vcb, vba) = 0. When (ρa, ρb) = 0, then from Eqs 9 we can obtain the integral solution for the temporal evolution of the variable Pc for a given arbitrary wc ≠ 0 and the initial conditions Pc = 0 at τ = 0 as follows.
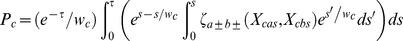
(14)When the TF gene C is turned on toward its maximum expression level then the input function 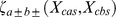 ∼ 1 and we can write the integral solution for temporal evolution of Pc under such conditions as follows.
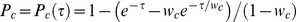
(15)From Eq 15 we find that 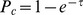 for wc is zero. Since the inequality 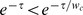 will be true for all the values of wc >0 and 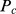 will tends toward zero as 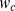 tends toward infinity, we can conclude that the response time associated with the expression of TF gene C upon induction of TF gene A will monotonically increase as wc increases. The maximum amount of TF protein C that is synthesized for a given rectangular pulse at the promoter of TF gene A with a width θ will be 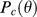 and one can measure the filtering efficiency of the FFL under consideration for an arbitrary pulse width θ >0 from the ratio 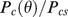. The cutoff pulse width θc can be defined by the inequality 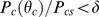 where δ is proportional to the experimentally detectable limit of the TF protein C under consideration. When wc = 0, then we find that 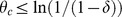 and when wc = 1 we find that 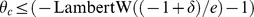 where LambertW(x) function is the solution of equation yey = x for y. Here we set δ ∼10−2 for simulation purposes. Upon substituting this value we find 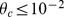 for wc = 0 and 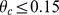 for wc = 1. For other values of wc, one needs to numerically solve the inequality 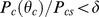 for θc. For example, when wc = 0.1 then we find that 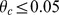. These results suggest that the critical cut-off pulse width θc increases along with the parameter wc.

### Stochastic Analysis

The set of Chemical Langevin equations (CLE) associated with the expression of TF genes A, B and C within the various types of A-OR-B type FFLs can be written as follows [26]–[28].

TF A:
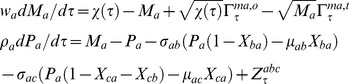
(16)


TF B:
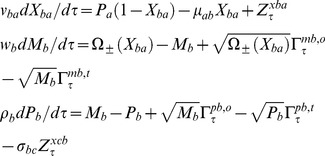
(17)


TF C:
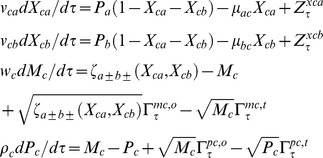
(18)


Eqs 16–18 suggest the presence of non-zero temporal correlations among the set of concentration variables 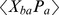, 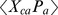 and 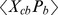. The characteristic correlation times associated with these pairs of variables will be much lesser than that of the timescales associated with the synthesis and decay of the respective protein products. This follows from the fact that the timescale associated with the promoter state fluctuations is well separated from the timescale associated with the synthesis and decay of the TF proteins. Here one should note that 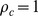 by definition. In this equation, various types of *Z* parameters are defined as follows.
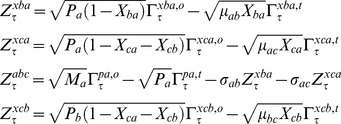
(19)In Eqs 16–19, the term
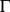
is the dimensionless delta-correlated Gaussian white noise with the following mean and variance properties.



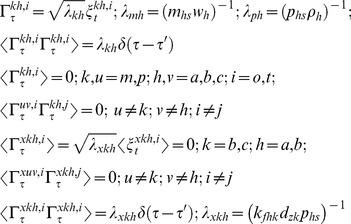
(20)In case of A-AND-B type FFLs there is an additional source of fluctuations arises from the dimerization reaction between the TF proteins A and B. The modified equations in Eqs 16–18 in the presence of A-AND-B type logic can be written as follows.
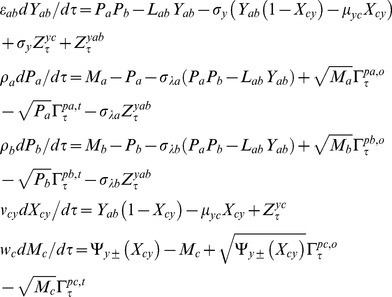
(21)In this equation, various types of *Z* parameters and the Gaussian noise terms 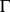 associated with the dimerization dynamics are defined as follows.
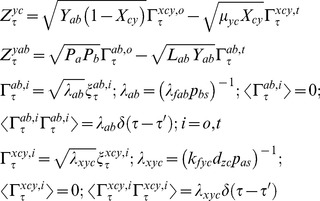
(22)


## Discussion

Response times of various type of FFLs are strongly dependent on the sets of parameters 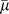, 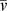, 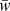 and weakly on 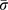. The sets of parameters 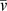 and 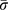 characterize the temporal coupling between the mRNA/protein dynamics and the binding-unbinding dynamics of protein molecules with the promoter. The ordinary perturbation parameter 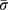 characterizes the strength of temporal coupling between the protein level dynamics with the promoter state occupancies. The variable 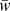 represents the ratios of the lifetimes of various mRNAs to lifetime of protein of TF gene C and reflects the coupling between the mRNA and protein degradation dynamics. The variable 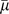, the set of dissociation constants is inversely proportional to the TFs/promoters binding affinity and characterizes the strength of various regulatory connections. The parameter set 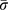 does not affect the rise-time of the TF gene C significantly since the change in the number of TF protein molecules due to binding-unbinding at various promoters is negligible. In contrast, 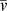 significantly affects the response times since the effect of varying 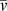 is indirectly amplified through the corresponding mRNA dynamics. Furthermore, an increase in 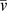 would decrease the rate at which promoter state occupancies shift towards saturation (in positive type regulation) or free form (in negative type regulation) as the regulatory TFs level builds up.

The parameter set 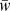 describes how best the rate of degradation of mRNAs of the TF genes A, B and C of FFLs are coupled to the rate of degradation of TF protein C. Lower values of 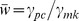 (*k = a, b, c*) occur only when the decay rate constants of various mRNAs (*γ_mk_*) are much higher than the decay rate (*γ_pc_*) of TF protein C. One should note that both the transcription and translation of various TF genes of prokaryotes are taking place in the cytoplasm whereas in case of eukaryotes the transcription is taking place inside the nucleus and the synthesized mRNA transcripts need to be spliced and then transported to cytoplasm through nuclear pores for translation. These differences in the cellular architecture warrants higher lifetimes (1/*γ_mk_*) for eukaryotic mRNAs than the prokaryotic ones which results in the general observation that the values of 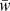 ( = lifetime of mRNA/lifetime of protein) associated with various genes in prokaryotes are lower than eukaryotes genes. It seems that a spectrum of various values of 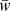 occurs in the protein coding genes of both prokaryotes and eukaryotes. In yeast, the values of 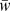 seems to vary from 0.1 to 1 with a median of ∼0.3 [17]–[19]. This means that we cannot ignore the dynamics of mRNAs while describing any type of TF network.

The parameter set 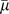 describes the strengths of various binding events associated with the FFLs under consideration. Higher values of 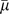 represent strong binding condition and lower values represent weak binding condition. Most of the interactions of TF proteins with the *cis*-acting DNA elements of associated promoters seems to be much stronger and 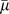 will be generally in the order of ∼10^−3^. When the steady state values of protein numbers in the absence of regulation under *in vivo* conditions is *p_ks_* ∼ 10^3^ then the value of 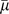
∼10^−3^ indicates that a single TF protein is enough to occupy the associated promoter for 50% of the observation times.

The parameter set 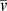 represents the strength of coupling between the promoter state occupancies and the rates of synthesis and degradation of TF proteins. The effect of 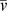 on the rate of synthesis and degradation of TFs will be generally mediated through the rate of changes in the respective mRNA levels. The speed at which a regulated promoter reaches its quasi-equilibrium state is inversely proportional to the value of 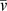. Higher values of 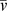 will slow down the rate at which promoter state occupancy reached its quasi-equilibrium state that in turn can increase the response times of positively regulated promoters and decrease the response times of negatively regulated promoter. Decrease in 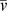 can also lead to overshooting of protein production in negatively auto regulated loops [9]. Small changes in 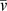 can significantly affect the dynamics of the associated TF protein since these changes are subsequently amplified by the dynamics of mRNAs. In most of the prokaryotic cases 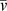 will be in order of ∼10^−4^ and it strongly dependent on the volume of the cell or nucleus in case of eukaryotic systems. Response times are not much influenced [9] by the parameter set 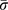 even in a wide range (10^−3^–10^3^). Here 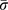 represents the direct coupling between the promoter state occupancies and the rate of synthesis and degradation of TF proteins. Variation in 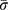 does not influence the response times much since the number of protein molecules associated with the binding-unbinding events are much less compared to the steady state values.

**Figure 2:**
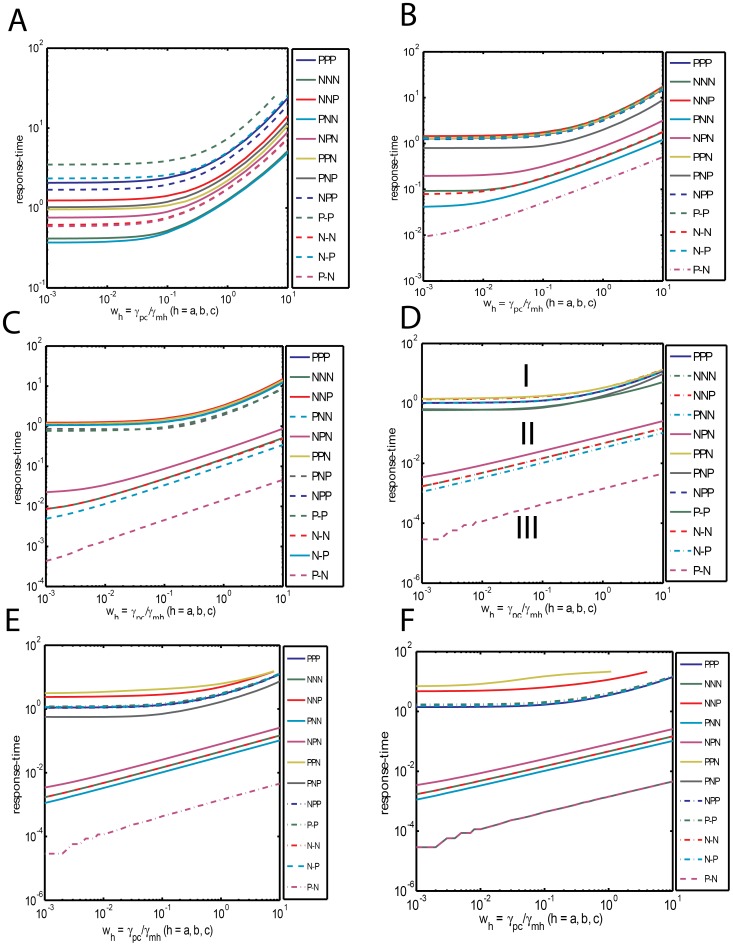
Dependency of response times on various sets of system parameters viz. 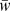
 = (*w_a_, w_b_, w_c_*), 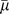 = (*µ_ab_, µ_ac_, µ_bc_, µ_yc_, L_ab_*) and 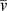 = (*v_ba_, v_ca_, v_cb_, ε_ab_, v_cy_*). Response times are expressed in terms of number of generation times. A. Dependency of response times of various types of FFLs under weak binding conditions. Here the simulation setting are 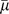 = 1, 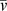 = 0.0003, 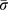 = 4, Δ*τ = 5* x10^−6^, the total simulation time was set to *T* = 25 generation times and 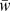 was iterated in the interval (0.001, 10) with Δ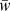 = 0.001. Under weak binding condition we observe that each of the considered FFLs behaves in a different way from others. B. Dependency of response times of FFLs 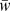. Settings are same as A with 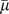 = 0.1. C. Dependency of response times of FFLs 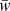. Settings are same as A with 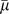 = 0.01. D. Dependency of response times of FFLs 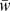. Settings are same as A however with 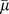 = 0.001. Under this strong binding conditions all the considered FFLs segregate into three Groups namely I, II and III. It seems that PPP (C1) and PNP (I1) type FFLs show less variation with respect to changes in 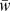 and also their response times are comparable with that of the unregulated C. E. Influence of increase in 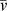 on the dependency of response times of FFLs on 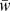. Here 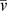 = 0.003. This is the physiological value of 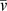 for a typical yeast cell nucleus whose volume is ∼10 times higher than a bacterial cell. In this case, P-P type FLL shifts from the third subgroup of Group I to the second subgroup. F. Influence of changes in 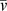 on the dependency of response times of FFLs on 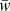. Here 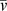 = 0.03. This is the physiological value for a typical human cell nucleus whose volume is ∼100 times larger than a bacterial cell. In this case, PNP (I1) type incoherent FFL shifts to Group III.

The dependency of the response times on the parameters 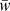 seems to be strongly influenced by the set of binding parameters 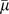. Under weak binding conditions such as 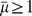, we find that each of FFLs under consideration show different type of variations as 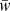 changes. Figure 2A suggests that the response times of various FFLs under weak binding conditions seems to be in the descending order of P-P, N-P, PPP, NPP, NNP, PNP, PPN, NPN, N-N, P-N, NNN and PNN. We further observe that almost all the FFLs show similar type of variation in response time with respect to changes in 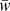 in the dynamic range 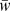 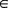 (0.1, 1). From Figures 2B, 2C and 2D we find that this scenario significantly changes as the binding strength increases. Under strong binding conditions such as 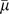 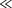
1, the entire set of FFLs can be approximately categorized into three subgroups (Figure 2D) based on the behavior of overall response times with respect to changes in 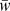 viz. Group I: {{PPN, NNP}, {PPP, NPP, N-P}, {P-P, PNP}}, Group II: {NPN, {N-N, NNN}, PNN} and Group III: P-N. Here the order of response times of various subgroups is Group I > Group II > Group III. One can write the segregation pattern in the standard terminology of FFLs as {{I3, C4}, {C1, I4, N-P}}, {P-P, I1}}, {C2, {N-N, I2}, C3}, P-N. Though Group II and Group III type FFLs possess lesser response times than Group I their response times increase almost linearly upon an increase 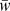 over the entire range of investigation on a log-log scale.

Within Group I we find three different subsets of FFLs having similar type of response times and their response times are in the order as {I3, C4} > {C1, I4, N-P} > {P-P, I1}. As we have pointed out in the introduction section, a FFL will be an efficient one when (1) the associated response times with respect to an input signal at the promoter of TF gene A is reasonably low or close to the generation time of the cell and (2) also it is less sensitive to the changes in 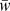 over the physiological dynamic range (0.1, 1). Those FFLs which satisfy these two criteria will be the efficient ones and naturally selected. Upon applying these two criteria on the subsets of Group I type FFLs, we find that C1 with both A-AND-B and A-OR-B gated logics and I1 are the preferred FFLs on overall range of 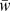 since response times of I3 and C4 type FFLs are higher than the generation time of the cell. We find from Figure 2D that the increase in the response times is <200% for Group I type FFLs upon increasing 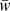 from 0.1 to 1 whereas it is >400% for Group II and III type FFLs. These results agree well with the earlier observations on the abundance of various FFLs in *E. coli* [10], [13] and yeast [10], [14]. Though Group II and III FFLs possess less response times, these FFLs are not selected by nature since the same speeding-up functionality can be achieved through a much simpler negative auto regulatory loops associated with the TF gene C [8]–[9].

Figures 2E and 2F suggest that the members of Groups I-III are not robust against variation of the parameter set 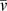 under strong binding conditions. Results show that the P-P type FFL will be moved from third subset of Group I to the second one upon an order of increase in 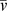. Further we find that the FFLs such as NPP, P-P, PPP and N-P behave in a similar way with respect to the changes in 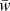 at higher values of 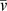. The segregation pattern of various FFLs based on the behavior of the response times over changes in 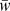 under such conditions seems to be {C4, I3, {C1, P-P, N-P, I4}, I1}, {C2, {N-N, I2}, C3}, P-N. Since the value 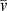 is inversely proportional to the speed at which the promoter state reaches the steady state, an increase in 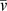 would decrease the transcription and translational rates. This will in turn increase the response times of positively regulated promoters and decrease the response times of negatively regulated promoters. Upon considering all these results together one can conclude that the FFLs of types PPP, P-P and PNP are robust against changes in both 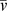 and 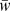. This also could be one of the reasons [14] associated with the observation [10] that these particular coherent C1 type FFLs with both OR and AND-logics are more abundant in nature than the other types. These results are summarized in Table 3.

**Table 3:** Summary of segregation patterns at weak and strong binding conditions.

Parameters	Segregation pattern of response times in 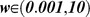	Abundances of various types of FFLsas in Ref [10]	Source
Condition I	P-P> N-P> C1> I4> C4> I1> I3> C2> N-N >P-N > I2> C3		
Condition II	{I3, C4} > {C1, I4, N-P} > {P-P, I1}, {C2> {N-N, I2} > C3}, P-N	C1> I1> C3> C2> {C4, I3, I4}	Prokaryotes
Condition III	{C4> I3} > {C1, P-P, N-P, I4} > I1, {C2> {N-N, I2} > C3}, P-N	C1> I1> C2> I2> I3	Eukaryotes (yeast)
Condition IV	{C4> I3} > {C1, P-P, N-P, I4}, {C2> {N-N, I2} > C3}, {P-N, I1}	I1 will be much lower than C1 type.	Higher eukaryotes

**Note**: This table summarizes the behavior of various FFLs under strong and weak binding conditions as well as fast and slow promoter-state dynamics. Under weak binding conditions (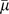 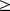
1) each FFL behaves differently from each other with respect to changes in 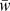. Here the settings for **Condition I**: weak binding and fast promoter state dynamics (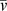 = 0.0003, 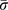 = 4, 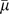 = 1). **Condition II**: strong binding and fast promoter state dynamics (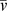 = 0.0003, 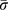 = 4, 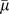 = 0.001). **Condition III**: strong binding and slow promoter state dynamics (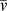 = 0.003, 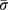 = 4, 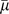 = 0.001). **Condition IV**: strong binding and slow promoter state dynamics (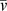 = 0.03, 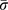 = 4, 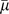 = 0.001). Under strong binding conditions 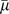 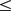
0.001, the entire set of FFLs segregates approximately into three subgroups I, II and III. Here P-P (C1 type FFL with AND type logic on TF gene C) and I1 behaves similarly and therefore the advantages of I1 type FFL whose response time is lower than the generation time will be shared by P-P type which will be added up to the C1 type FFL with OR type gated logic to TF gene C. However this pattern seems to be weakly dependent on 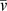. When 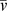 increases as in case of eukaryotic cell, then P-P behaves similar to C1 type and I1 type FFL will have the entire advantage of having lower response times than other subgroups of Group I. As a result, I1 type FFL will be more abundant in eukaryotes than prokaryotes. All these results are not dependent on changes in 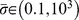, increasing 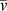 beyond 0.03 or decreasing below 0.0003. One should note that the physiological value of 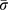 in prokaryotes will be 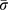 ∼ 4. The overall response time of first subgroup (I3 and C4) of Group I is higher than the generation time of the cell. The response times of the second subgroup (C1, I4 and N-P) are closer to the generation time whereas the third subgroup possess lesser response times than the others. The response times of the FFLs in Group-I are more robust against changes in 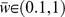 over the physiological values than Group II and III. Comparison with the relative abundances of naturally occurring FFLs, one can conclude that those FFLs are naturally selected when their response times are (a) robust against changes in 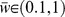 and (b) closer to or lesser than the generation time.

**Figure 3:**
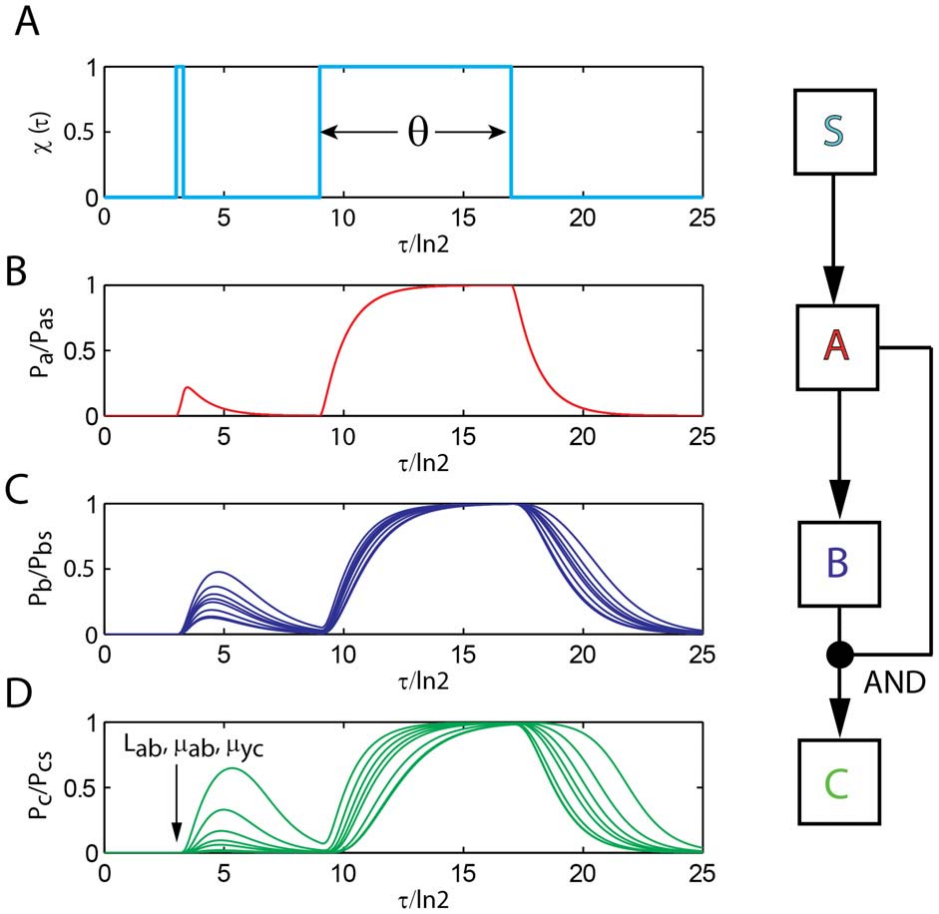
Dependency of filtering efficiency of P-P type (C1 type with AND-logic) FFL on the set of binding parameters 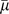
 **.** Here the general settings are: 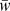 = 0.12, 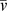 = 0.0003, 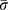 = 4, *T* = 25 generation times, and 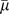 was varied as (0.1, 0.2, 0.3, 0.4, 0.5, 1, 3 and 5). A. Input signal at the promoter of TF gene A. This has one short rectangular pulse with a width of *θ = *0.3 and a large one with *θ = *8 all are measured in terms of number of generation times. B. TF gene A responds similarly for all values of 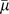 to both the signals irrespective of the binding strengths. The response seems to be proportional to the pulse width *θ* without any delay. C. As binding strength increase, the response of B also increases proportionately. D. There exists a cutoff value of 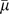 above which the expression level of TF gene C is practically zero. With the current settings, this cutoff seems to occur at 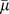 = 1. Arrow shows the increasing direction of 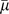.

**Figure 4:**
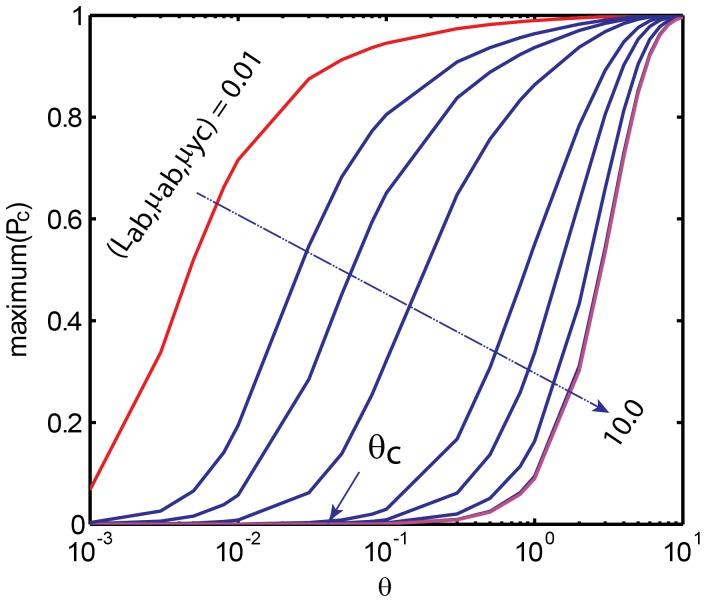
Dependency of the maximum achievable protein product of TF gene C on the width of the input pulse at the promoter of A in case of P-P A-AND-B type FFL (C1 with AND-logic). Here the settings are 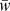 = 0.12, 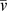 = 0.0003, 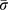 = 4, *T* = 25 generation times, and 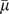 was varied as (0.01, 0.03, 0.05, 0.1, 0.3, 0.5, 1, 5 and 10). Beyond 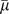
>5, there is not much change in the variation of maximum of *P_c_* with respect to the pulse width. The cutoff pulse width *θ_c_* seems to be strongly dependent on the binding parameters 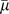.

When the binding parameters (*µ_ab_, µ_yc_, L_ab_*) are much lesser than one, then we find from Eq 11 that the TF gene C will be turned on toward its maximum expression level. This means that strong binding conditions are required for the following types of molecular interactions to achieve the maximum production of TF protein C viz. (1) protein-protein interactions between A and B, (2) binding of the dimer A–B at the promoter of C and (3) binding of protein A at the promoter of B. Conditions (1–3) also suggest that for an efficient filtering activity of the P-P type A-AND-B FFL against transient input signals at the promoter of the TF gene A, the inequality conditions (*µ_ab_, µ_yc_, L_ab_*) 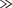 1 are necessary which in turn will decrease the maximum achievable steady state value of protein C as shown in Figure 3. In other words there exists a critical value of 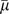 = 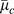 for a given signal with a pulse width *θ* above which the response of gene C for a pulse of signal at the promoter of gene A will be practically zero. In Figure 3, this critical value occurs at 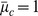 for a pulse width of *θ = *0.3. On the other hand, for a given value of 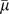 there exist a cutoff pulse width *θ = θ_c_* below which the response of TF gene C is practically zero which is demonstrated in Figure 4. From Figure 4 we find that there also exists a transition region in which the TF gene C responds to the width of the input pulse that is given at the promoter of A in a graded manner. From Eqs 10 we find that 
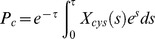
 and when 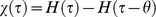, then we can conclude that the TF gene C will respond to the variation of width *θ* of the activation signal in a graded manner in the strong binding limit as (*µ_ab_, µ_yc_, L_ab_*) tend toward zero. The maximum achievable *P_c_* will increase proportional to the width of the activation signal. Whereas in the weak binding limit as the binding parameters (*µ_ab_, µ_yc_, L_ab_*) tend toward infinity, the response of TF gene C with respect to the pulse width *θ* seems to be approximately a sharp type. Under such conditions there exists a critical pulse width (*θ_c_*) with a sharp transition region above which the maximum achievable *P_c_* will be *P_c_* ∼ 1 and below which the maximum achievable *P_c_* will be *P_c_* ∼ 0.

**Figure 5:**
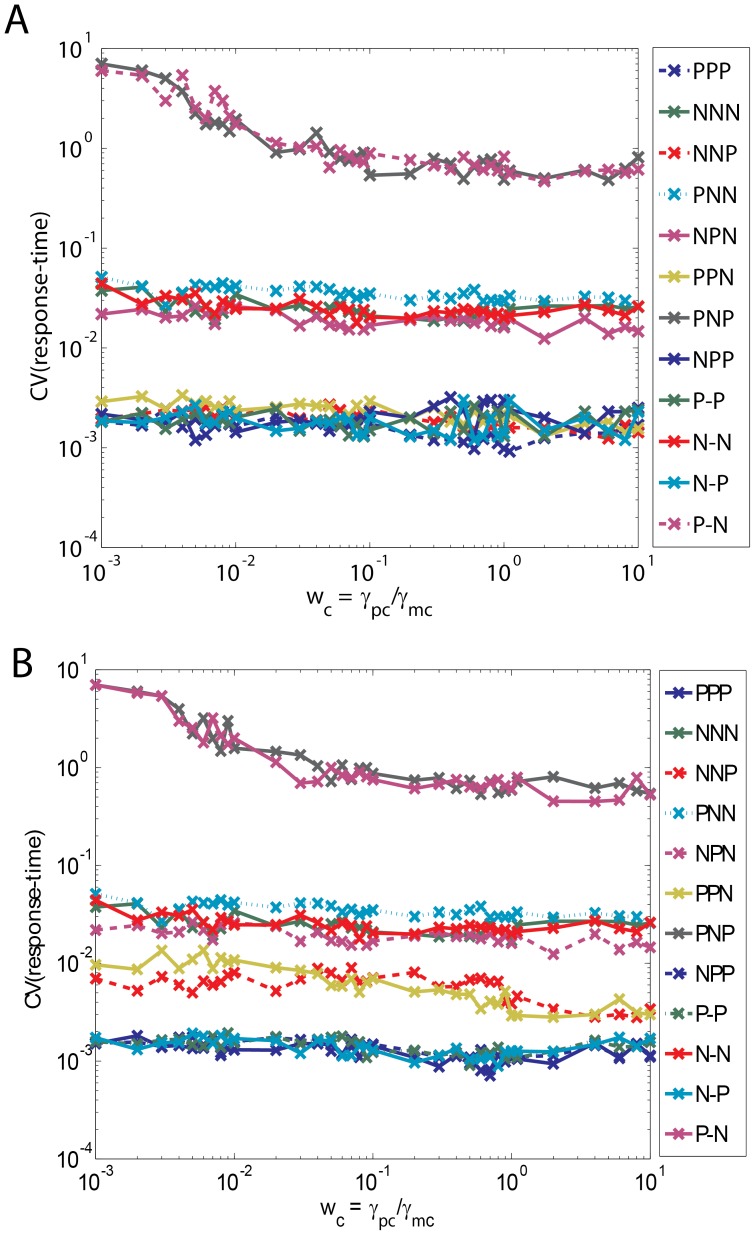
Coefficient of variation (CV) associated with the fluctuations in the response times of various types of FFLs under strong binding conditions ( 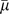
 **= 0.001).** CV was calculated over 10^5^ numbers of stochastic trajectories. The coherent C1 type FFL possesses lower CV of response times than other FFLs whereas I1 type possesses highest CV of response times. A. Here the settings are 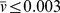 that is applicable to both prokaryotes and eukaryotes such as yeast, 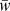 was iterated from 0.001 to 10, and 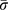 = 4, *T* = 25 generation times. B. Here the settings are 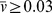 that is applicable to higher eukaryotes such as human, 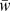 was iterated from 0.001 to 10, and 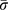 = 4, *T* = 25 generation times.

It seems that the parameters (*θ_ab_, θ_yc_, L_ab_*) associated with various binding events need to be fine-tuned to achieve both high efficiency in the filtering activity as well as maximum possible steady-state values of protein *P_c_* upon inducing the promoter of gene A by a persistent signal. Results from stochastic simulations at various values of 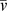 and *w_c_* are shown in Figure 5 and summarized in Table 4. These results suggest that the coefficient of variation in the response times associated with various FFLs under strong binding conditions (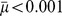) are robust against changes in *w_c_* as well as 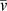. When 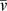
<0.003 (as in case of prokaryotes and yeast), then based on the overall coefficient variation in the response times all the FFLs can be categorized into at least three different groups viz. {I1, P-N} > {C3, N-N, C2, I2} > {C1, C4, I3, P-P, N-P, I4}. When 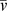
>0.003 (as in case of higher eukaryotes such as human and plants), then the segregation pattern associated with the overall coefficient of variation in the response times seems to be as {I1, P-N} > {C3, N-N, C2, I2} > {C4, I3} > {C1, P-P, N-P, I4}. The coefficient of variation in the response time of the first group seems to be >100% and in the second group it is 1% and in the third/fourth group of FFLs it is 0.1%. The coherent C1 FFL with both OR/AND type logic shows the least amount of variation in the response times among all the FFLs under consideration.

**Table 4:** Segregation patterns of CV (response time) at weak and strong binding conditions.

Parameters	Segregation pattern of CV (response-time) in 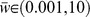	Abundances of various typesof FFLs as given in Ref [10]	Source
Condition II	{I1, P-N} > {C3, N-N, C2, I2} > {C1, C4, I3, P-P, N-P, I4}	C1> I1> C3> C2> {C4, I3, I4}	Prokaryotes
Condition III	{I1, P-N} > {C3, N-N, C2, I2} > {C1, P-P, C4, I3, I4, N-P}	C1> I1> C2> I2> I3	Eukaryotes (yeast)
Condition IV	{I1, P-N} > {C3, N-N, C2, I2} > {C4, I3} > {C1, P-P, N-P, I4}	I1 will be lower than C1 type.	Higher eukaryotes

**Note**: This table summarizes the results from the stochastic simulation. Parameter settings for **Condition II**: strong binding and fast promoter state dynamics (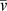 = 0.0003, 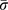 = 4, 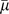 = 0.001). **Condition III**: strong binding and slow promoter state dynamics (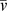 = 0.003, 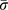 = 4, 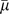 = 0.001). **Condition IV**: strong binding and slow promoter state dynamics (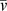 = 0.03, 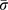 = 4, 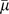 = 0.001). Here CV (defined as the ratio standard deviation/mean) represents the coefficient of variation in the response times (the time required to attain half of the steady state value of the transcription factor protein C in the FFLs).

Earlier results ([10], supplementary materials) based on the analysis of literature-based databases of experimentally verified direct transcription interactions for *E. coli* [6] suggested a distribution pattern of various FFLs as 83% coherent (out of which 80% were C1 type, 11% C3, 6% C2 and 3% C4) and 17% incoherent (out of which 72% were I1, 14% I2 and 14% I4) types. Similar analysis on *S. cerevisiae* [6], [10] showed a distribution of FFLs as 55% coherent (out of which 84% were C1 and 16% C2) and 45% incoherent (out of which 84% were I1, 12% I2 and 4% I3) types. This data suggested an overall distribution of various FFLs in the transcription factor networks of bacteria as (67% C1, 12% I1, 10% C3, 5% C2, 1% C4, 1% I3 and 1% I4). The overall distribution of various FFLs in the TF networks of yeast is (47% C1, 38% I1, 9% C2, 5% I2 and 1% I3). Comparison of the order of preferences of various FFLs in bacteria (C1> I1> C3> C2> {C4, I3, I4}) and yeast (C1> I1> C2> I2> I3) with the Group I, II and III types of FFLs, one can conclude that those FFLs of Group I (C1, I1) whose response times are close to or less than the generation time of the cell apart from the robustness against changes in 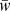 are more preferably selected. The next set of preferable FFLs (C2, C3 and I2) is from Group II. The set of FFLs (I3, I4 and C4) are the least preferable ones. Although I3 and C4 fall within Group I, their response times are much higher than the generation time. It seems that the relative abundance of I1 type FFL is more in eukaryotes than the prokaryotes. Here one should note that the value of the parameter set 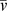
(*v_hk_ = γ_pc_/k_fhk_p_hs_* = 0.0003) that is used in our simulations (Fig. 2A–D) is derived for a prokaryote organism whose cell volume is ∼10^−18^ m^3^. With this setting we find from our simulations that the subset (P-P and I1) behaves in a similar way within Group I which is evident from the segregation pattern of various FFLs at strong binding conditions {{I3, C4}, {C1, I4, N-P}, {P-P, I1}}, {C2, {N-N, I2}, C3}, P-N. We argue that some of the advantages of I1 such as lesser response time than the generation time apart from the robustness against changes in 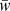 will be shared by P-P type FFL that is in turn added to the total abundances of C1. This follows from the fact that P-P is also a C1 type FFL with AND gated logic type regulation on the promoter of TF gene C. Stochastic simulations results suggested that the coefficient of variation in the response times is lesser in C1 type FFLs than the other FFLs which agrees well with the observation that these types are relatively more abundant in nature. On the other hand I1 type FFLs possess highest coefficient of variation in the response times. Overall results suggest that the coefficient of variation in the response times is relatively higher whenever the type of regulation imparted by TF genes A and B on the TF gene C is negative. This result is in line with the observations on the negatively self-regulated TF networks in which the coefficient of variation in the response times was shown [9] to be >50%. These results suggest that I1 type FFL as well as negative self-regulated motifs can speed up the response time at the cost of increasing the fluctuations in their response times.

Since the volume of eukaryotic nucleus is much larger than a bacterial cell volume over several orders of magnitudes (volume of yeast nucleus is ∼10^1^ times larger than a bacterial cell volume [25] whereas human cell nucleus is ∼10^2^ times larger than a bacterial cell volume), the overall bimolecular collision rate associated with the binding of TFs at their cognate sites (*k_fgh_*) inside the nucleus will be much lower in case of eukaryotes than prokaryotes owing to the dilution effects. This means that the physiological values of 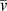 will be higher in eukaryotes than prokaryotes. Upon an increase in 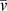 as in case of eukaryotes such as yeast ( = 0.003), P-P type behaves in a similar way (Fig. 2E–F) as that of C1 whereas I1 type FFL recovers the full advantage over its lower response time within Group I compared to C1 which is evident from the altered segregation pattern {C4, I3, {C1, P-P, N-P, I4}, I1}, {C2, {N-N, I2}, C3}, P-N. This in turn will result in the higher relative abundances of I1 type FFL in eukaryotes than the prokaryotic TF networks. Further increase in 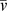 as in case of nucleus of human cell ( = 0.03) shift the I1 type FFL from Group I to Group III. This result predicts that I1 type FFLs will be much lesser in abundance than the C1 type in the TF network of human and other higher animals and plants. One also should note that we are still not able to explain the relative scarcity of I4 and N-P type FFLs even though they fall well within Group I. As pointed out by Magnan and Alon in reference [10], the relative scarcity could be due to the reduced functionality of type 3 and type 4 FFLs with AND gated logic (here it is applicable to N-P) since they response at most one of the triggering signals given at the promoters of both the genes A and B.

We have considered the sets of similar parameters (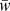, 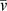, 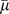, 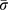, 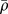) associated with TF genes A, B and C as variable units rather than individual ones for all these calculations and simulations. The TF network of an organism consists of several FFL motifs and each of these FFLs is constituted with different subsets of a pool of TF genes. Each subset of TFs can be represented as points in the parametric space of our model. Upon considering the entire pool of TF genes, one should note that each of the members of these sets of parameters can take a spectrum of values with a probability distribution with definite mean and variance. There are two types of variability of parameters among the TF genes of FFLs viz. variation of the parameters of TF gene A/B/C across various FFLs found in the entire TF network and variation of the parameters of TF genes A, B, C within a given FFL. Here we have assumed that both of them are approximately the same. The main results of our analysis will not be affected much due to the second type of variation since we have used the mean values of the parameters associated with the entire set of TF genes of an organism to represent genes A, B and C of various types FFLs.

## Methods

We use the following Euler type iterative numerical scheme to integrate the deterministic differential Eqs 1–3 associated with various A-OR/AND-B type FFLs in the dimensionless time and concentrations space.

TF A:

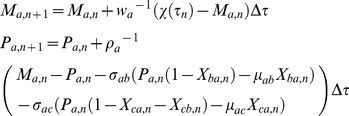
(23)


TF B:

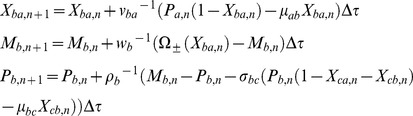
(24)


TF C:

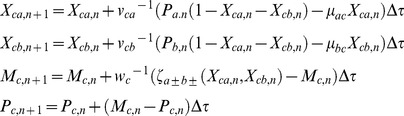
(25)


To investigate the filtering efficiency of P-P type A-AND-B FFL we can use the signal function 
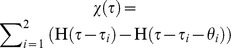
 at the promoter of TF gene A with two well separated rectangular pulses with respectively lower and higher pulse widths. For the purpose of computing the response times we can reset the input function at the promoter of gene A as 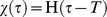 where *T* is the total simulation time. The numerical scheme for the modified Eqs 6–8 for *Y_ab_, P_a_, P_b,_ X_cy_,* and *M_c_* in case of A-AND-B type FFLs can be written as follows.
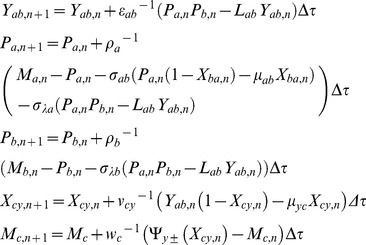
(26)


The total scaled simulation time *T* will be divided into *N* equal intervals such that Δ*τ = T/N.* For simulation purpose we set Δ*τ = *5×10^−5^ and the corresponding Δ*t = *0.2s for a lifetime 1/*γ_pc_* ∼ 60 mins. The initial conditions are *X_hg,0_* = 0, *M_h,0_* = 0 and *P_h,0_* = 0 for *h = a, b, c* and *g = b, c.* To compute the response-time of the TF gene C we set an absorbing boundary at *P_c_/P_cs_ = *1/2 where *P_cs_* is the steady state value of scaled concentration *P_c_* which in turn depends on the type of FFL under consideration as given in Eq 13. We measure the concentrations in terms of number of molecules inside the cell. Considering a bacterial cell (volume∼10^−18^ m^3^) [21] we set 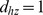, 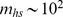 molecules and 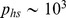 molecules [22], [23] where *h = a, b* and *c* respectively denotes TF genes A, B and C. Concentration of a single TF molecule inside a bacterial cell will be ∼2 nM. Concentration of a single TF molecule inside the nucleus of yeast cell [24], [25] will be ∼200 pM and inside the nucleus of human cell it is ∼20 pM. We measure the timescales in terms of the lifetime of the TF protein C by dividing *t* by *γ_pc_*. Since the dynamics of binding-unbinding of the transcription factors with the respective promoters is a typical diffusion-controlled site-specific DNA-protein interaction, under *in vivo* conditions of bacterial cell we find that 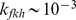 molecules^−1^s^−1^ [22]–[24]. Here we have assumed an in vivo three dimensional diffusion controlled collision rate ∼10^6 ^M^−1^s^−1^. In case of nucleus of yeast cell we find 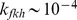 molecules^−1^s^−1^ and in case of nucleus of human cell we find 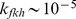 molecules^−1^s^−1^. Here the TF protein *k* = (*a, b*) binds with the promoters of *h* = (*b, c*). It will be a complicated task to explore the entire parametric space. To simplify the analysis further we can consider the sets of parameters viz. 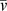 = (*v_ba_, v_ca_, v_cb_, ε_ab_, v_cy_*), 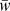 = (*w_a_, w_b_, w_c_*), 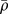 *= *(*ρ_a_, ρ_b_*), 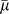 = (*µ_ab_, µ_ac_, µ_bc_, µ_yc_, L_ab_*) and 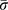 = (*σ_ab_, σ_bc_, σ_ac_, σ_λs_, σ_λb_*) as the parametric units of our numerical simulations. When all the TF proteins A, B and C decay with similar decay rate constants then we find that 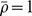. Using the steady state values of various proteins and mRNA numbers in the absence of any regulation one obtains 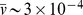
(∼0.003 for nucleus of yeast cell and ∼0.03 for nucleus of human cell) and 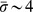 (∼0.4 for yeast cell nucleus and ∼0.04 for nucleus of human cell) where we have used a protein decay rate *γ_pc_* ∼ 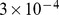
s^−1^ (for a protein lifetime of ∼60 min) to transform the real time variables to *τ* space variables. The dependency of various properties of TF gene C such as response-time, delay with respect to the induction signal at the promoter of gene A and maximum achievable *P_c_* on the parameter sets 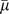, 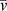, 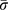 and 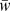 were explored. The values of 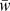 seems to vary across the spectrum of genes from 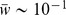 in case of prokaryotes to 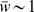 in case of eukaryotes [17]–[19]. Together with all these values we iterated the parameter set 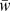 inside the range (0.001, 10) with a step size of 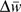 = 0.001. For further exploratory purposes we considered four different binding conditions from low to high viz. 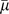 = (0.001, 0.01, 0.1, 1) and three different values of 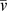 = (0.0003, 0.003, 0.03).

Similar to Eqs 21–24 we use Euler type numerical scheme to integrate the Chemical Langevin Eqs 16–20 where we replace 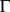
with Gaussian distributed random numbers with zero mean and unit variance. Assuming an overall *in vivo* steady state mRNA levels as *m_hs_* ∼ 10^2^ and proteins levels as *p_hs_* ∼ 10^3^ (where *h* = *a, b, c*, measured in number of molecules) we find that *λ_mh_* ∼ 10^−2^/*w_h_* molecule^−1^ and *λ_ph_* ∼ 10^−3^/*ρ_h_* molecule^−1^. Further we also find that *λ_ab_* ∼ 1 s, *λ_xkh_* ∼ 10^−3^ molecule^−1 ^s (*k* = *b,* c and *h = a*, *b*) and *λ_xyc_* ∼ 10^−3^ molecule^−1 ^s. We use the reflecting boundaries (0, 1) for the scaled concentration variables (*P_h_, M_h_, Y_ab_,* and *X_nk_*) where *n = b, c* and *k = a, b* and the absorbing boundary condition *P_c_* = ½ to compute the mean first passage time (response-time) from the stochastic simulations. Averaging was done over 10^5^ trajectories at each *w_c_* value and the coefficient of variation of response time was computed as CV (response-time) = standard deviation of response-time/mean of response-time.

### Conclusions

Feedforward loops (FFLs) consist of three genes which code for three different transcription factors A, B and C where B regulates C and A regulates both B and C. We have developed a detailed model to describe the dynamical behavior of various types of coherent and incoherent FFLs in the transcription factor networks. We considered the deterministic and stochastic dynamics of both promoter-states and mRNAs of various genes coding for the transcription factors associated with the FFL motifs. Detailed analysis showed that the response times of FFLs strongly dependent on the ratios (*w_h_* = *γ_pc_/γ_ph_* where *h = a, b, c*) between the lifetimes of mRNAs of A, B and C (1/*γ_mh_*) and the protein of C (1/*γ_pc_*). When the binding of transcription factors A and B with the *cis*-acting elements of respective promoters is very strong, then we could categorize all the possible FFLs into Group I, II and III based on the dependency of the response times of FFLs on *w_h_*. Though the response times of Group I FFLs were higher than II and III, they seem to be less sensitive to the changes in *w_h_* within the naturally occurring dynamic range (0.1, 1). We have further shown that among the members of the Group I FFLs, the coherent C1 type was more robust against changes in other system parameters which could be one of the reasons why C1 type coherent FFLs are more abundant in nature than the others.
